# DNA Methylation and Gene Expression Changes in Monozygotic Twins Discordant for Psoriasis: Identification of Epigenetically Dysregulated Genes

**DOI:** 10.1371/journal.pgen.1002454

**Published:** 2012-01-19

**Authors:** Kristina Gervin, Magnus D. Vigeland, Morten Mattingsdal, Martin Hammerø, Heidi Nygård, Anne O. Olsen, Ingunn Brandt, Jennifer R. Harris, Dag E. Undlien, Robert Lyle

**Affiliations:** 1 Department of Medical Genetics, Oslo University Hospital and University of Oslo, Oslo, Norway; 2 Research Unit, Sorlandet Hospital, Kristiansand, Norway; 3 Institute of Psychiatry, University of Oslo, Oslo, Norway; 4 Department of Dermatology, Oslo University Hospital and University of Oslo, Oslo, Norway; 5 Division of Epidemiology, Norwegian Institute of Public Health, Oslo, Norway; Queensland Institute of Medical Research, Australia

## Abstract

Monozygotic (MZ) twins do not show complete concordance for many complex diseases; for example, discordance rates for autoimmune diseases are 20%–80%. MZ discordance indicates a role for epigenetic or environmental factors in disease. We used MZ twins discordant for psoriasis to search for genome-wide differences in DNA methylation and gene expression in CD4^+^ and CD8^+^ cells using Illumina's HumanMethylation27 and HT-12 expression assays, respectively. Analysis of these data revealed no differentially methylated or expressed genes between co-twins when analyzed separately, although we observed a substantial amount of small differences. However, combined analysis of DNA methylation and gene expression identified genes where differences in DNA methylation between unaffected and affected twins were correlated with differences in gene expression. Several of the top-ranked genes according to significance of the correlation in CD4^+^ cells are known to be associated with psoriasis. Further, gene ontology (GO) analysis revealed enrichment of biological processes associated with the immune response and clustering of genes in a biological pathway comprising cytokines and chemokines. These data suggest that DNA methylation is involved in an epigenetic dysregulation of biological pathways involved in the pathogenesis of psoriasis. This is the first study based on data from MZ twins discordant for psoriasis to detect epigenetic alterations that potentially contribute to development of the disease.

## Author Summary

Psoriasis is a common chronic inflammatory disease, which affects mainly the skin, but also the joints. It is considered a T cell–mediated autoimmune disease. Autoimmune diseases are in general due to a dysregulation of the immune system, and identification of genes involved in alterations of lymphocyte function is therefore essential. Although there is convincing evidence of a genetic basis underlying psoriasis, there is also evidence for the involvement of environmental and/or epigenetic factors. Here we use MZ twins discordant for psoriasis to search for disease-causing epigenetic and transcriptomic alterations in isolated lymphocyte subpopulations. We identified many genes involved in the immune response where changes in DNA methylation between unaffected and affected twins correlated with changes in gene expression. In addition, several of these genes had previously been identified in GWAS and linkage studies of psoriasis. These findings suggest that psoriasis involves an epigenetic dysregulation of immune-system genes.

## Introduction

Psoriasis is a common chronic inflammatory disease, which affects mainly the skin, but also the joints. The worldwide prevalence is reported to range between 1–11.8% depending on ethnicity, geographical area and method of assessment [1]. Psoriasis is known to have a strong genetic component with an estimated heritability of 66% [2]. Linkage peaks [3], copy number variations (CNVs) [4]–[6] and genes identified by GWAS [7]–[13] are associated with psoriasis. However, the combined effect of these loci does not account for the genetic variation underlying the observed susceptibility to psoriasis, and indicates the involvement of additional genetic, epigenetic or environmental factors [7], [14]–[15]. Further evidence for the role of epigenetic or environmental factors comes from the fact that the concordance rate among MZ twins is only 35–72% [2], [14], [16].

Phenotypic differences are a result of genetic and epigenetic variation, as well as environmental influences. The study of discordant MZ twins provides an attractive model to investigate epigenetic mechanisms in disease. Environmentally driven epigenetic changes are thought to contribute to development of autoimmune diseases through alteration of epigenetic profiles, but exactly how the environment modulates epigenetic states is not well understood. Besides twin discordance, accumulating evidence supports the contribution of epigenetics in the development of autoimmune diseases through dysregulation of the immune system [17]–[20]. Psoriasis is considered a T cell-mediated autoimmune disease, and T cell activation is a key event in the pathogenesis [21]. Antigen binding results in a complex downstream signaling pathway in which epigenetic mechanisms have an important role [21], [22]. A likely scenario involves an abnormal activation and migration of T cells into the skin, followed by a cascade of events, which ultimately results in aggregation of the inflammatory cells and development of psoriasis. The development of psoriatic plaques is a result of an activation of cells in focal skin regions, which is mediated by CD4^+^ (helper) and CD8^+^ (cytotoxic) cells [23].

The aim of this study was to identify epigenetically dysregulated genes, which contribute to development of psoriasis. To do this, we assessed the extent of epigenetic and transcriptomic differences in CD4^+^ and CD8^+^ cells isolated from MZ twins discordant for psoriasis. This study design enabled identification of cell-type specific DNA methylation differences which correlate with gene expression, and thereby identification of genes where DNA methylation may have a functional role in development of psoriasis.

## Results/Discussion

**Figure 1:**
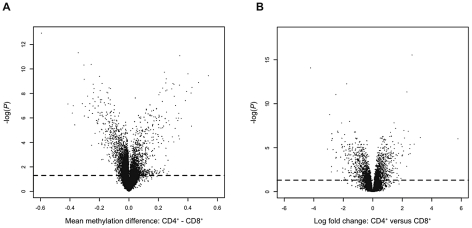
Volcano plots of cell-type specific differences in DNA methylation and gene expression. (A) Differences in DNA methylation between CD4^+^ and CD8^+^ cells. Each point represents a CpG site, with mean β-value difference across 12 unaffected individuals along the *x*-axis and −log10 of a corrected *P*-value from a paired *t*-test along the *y*-axis. (B) Differences in gene expression between CD4^+^ and CD8^+^ cells. Each point represents a gene, with mean log2 fold change across 13 unaffected individuals along the *x*-axis and −log10 of a corrected *P*-value from a paired *t*-test along the *y*-axis. Dashed lines represent the FDR of 5%.

Since genome-wide patterns of DNA methylation are known to differ between cell-types [24]–[26], and different cell-types in the immune system are implicated in the pathogenesis of psoriasis, we isolated and studied CD4^+^ and CD8^+^ cells to overcome the issue of epigenetic heterogeneity in whole blood. Both cell-types are relatively abundant in blood and have important functions in the immune system, thus they are good targets for epigenetic alterations which might contribute to the development of psoriasis. Comparisons of CD4^+^ and CD8^+^ cells revealed many significant differences for both DNA methylation (1,288 of the 26,690 CpG sites, 4,8%, *n* = 12, Table S1) and gene expression (2,126 of the 37,846 transcripts, 5,6%, *n* = 13, Table S2) in unaffected individual twins (Figure 1), which clearly demonstrate the importance of isolating specific cell-types.

**Figure 2:**
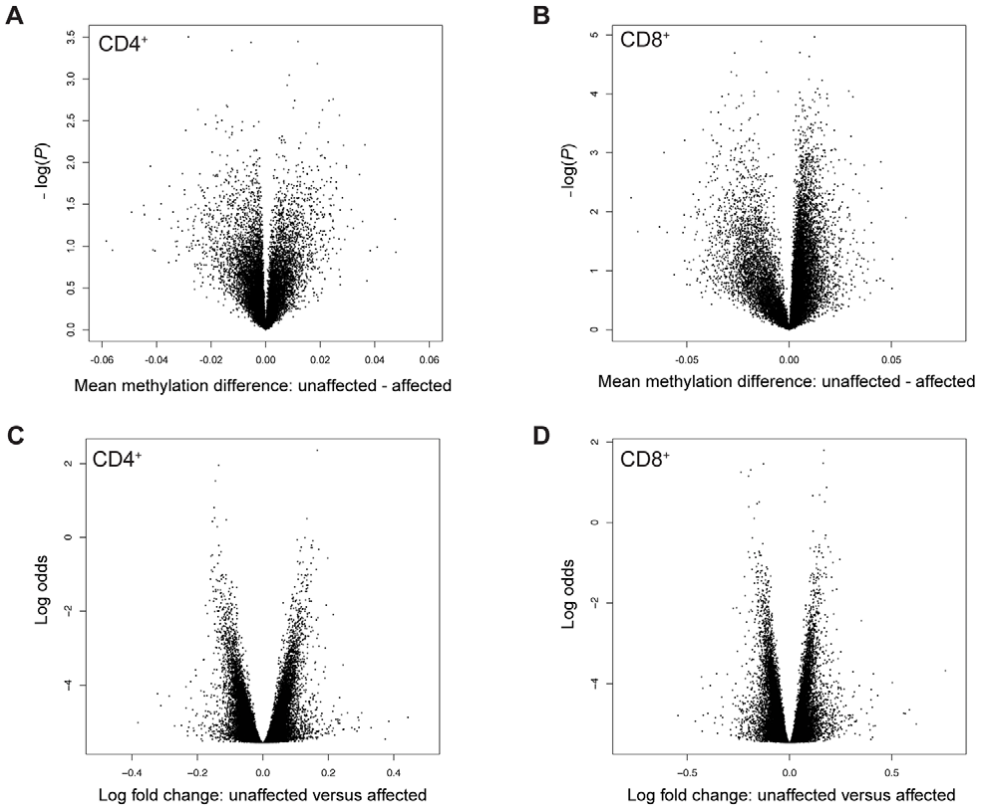
Volcano plots of differences in DNA methylation and gene expression in discordant MZ twins. (A–B) Differences in DNA methylation in CD4^+^ (*n* = 17 pairs) and CD8^+^ cells (*n* = 13 pairs), respectively. Each point represents a gene, with mean co-twin β-value difference along the *x*-axis and −log10 of the uncorrected *P*-value from a paired *t*-test along the *y*-axis. (C–D) Differences in gene expression in CD4^+^ cells (*n* = 17 pairs) and CD8^+^ cells (*n* = 14 pairs), respectively. Each point represents a gene, with mean log2 fold change along the *x*-axis and log odds along the *y*-axis.

MZ co-twins are highly correlated for DNA methylation in both CD4^+^ cells (*n* = 17 pairs, mean *ρ* = 0.98, range = 0.96–0.99) and CD8^+^ cells (*n* = 13 pairs, mean *ρ* = 0.97, range = 0.95–0.98) (Figure 2A and 2B). Both analyses of individual CpG sites and mean methylation per gene did not reveal any differentially methylated sites between unaffected and affected co-twins. Individual scatter plots of DNA methylation clearly demonstrate greater similarity among the MZ twins than among unrelated individuals (Figure S1). To ensure that the observed differences in DNA methylation between co-twins were genuine rather than technical artifacts, we ran internal replicates on a subset of the twins. Specifically, we replicated 7 pairs and calculated technical and biological differences between replicated samples (self-self comparisons) and between co-twins, respectively. The overall distributions of the technical differences in DNA methylation per CpG site were significantly smaller than the biological differences (Kolmogorov-Smirnov test, two-sided, *P*-value<2.2×10^−16^, Figure S2). This clearly shows that the observed biological differences between unaffected and affected co-twins are genuine, although they are small. Similarly, differences in gene expression between MZ co-twins were small in both CD4^+^ cells (*n* = 17 pairs, mean *r* = 0.99, range = 0.97–0.99) and CD8^+^ cells (*n* = 14 pairs, mean *r* = 0.99, range = 0.98–0.99). Although there are many small differences, we did not detect any genome-wide significant differences in DNA methylation or gene expression between co-twins discordant for psoriasis when analyzed separately (Figure 2C and 2D).

**Figure 3:**
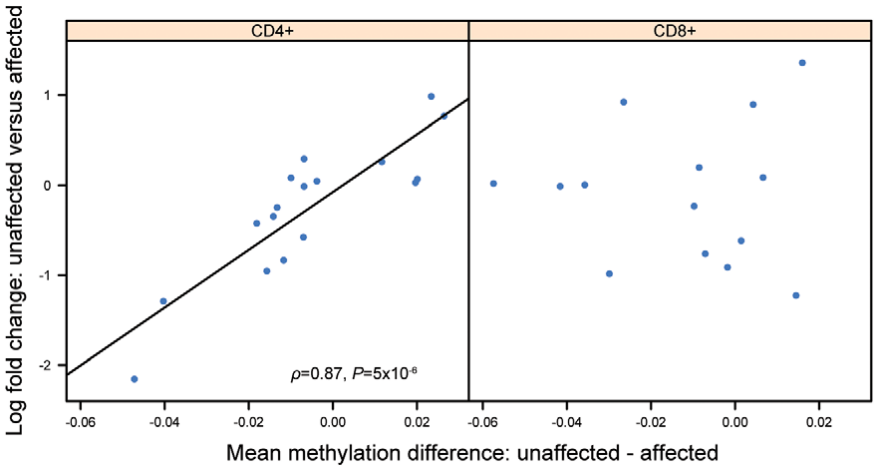
Scatter plots of differences in DNA methylation against the differences in gene expression of TNFSF11. The plots show a correlation of DNA methylation differences and gene expression differences in MZ co-twins. Each point represents a twin pair, with the mean difference in DNA methylation β-value (of 2 CpG sites) along the *x*-axis, and log2 fold change along the *y*-axis. A correlation of 0.87 was calculated in CD4^+^ cells.

DNA methylation is essential for the regulation of gene expression. We reasoned that a combined analysis of DNA methylation and gene expression could select functional methylation sites involved in regulating gene expression. We therefore investigated if co-twin differences in DNA methylation and gene expression were correlated. To do this, we compared the differences (between co-twins) in mean β-values for the CpGs associated with each gene, with the log fold changes of the gene expression. Using Spearman's rho as a measure for correlation, we then ranked the genes according to the significance of the correlation coefficients. This combined analysis of DNA methylation and gene expression revealed cell-type specific differences, identifying genes known to be involved in immune response and associated with psoriasis, only in CD4^+^ cells. Table 1 shows the top 50 genes ranked according to the significance of the correlation in CD4^+^ cells. Genes associated with psoriasis (shown in bold) are overrepresented in this list (Fisher's exact test, *P* = 3.3×10^−5^, Table S3). IL13 have been identified in GWAS [8], [10], [11], [27], and *ALOX5AP* [28], *PTHLH* [29] and TNFSF11 [30] have all been linked to different aspects of the disease. The entire list of the 11,933 genes studied ranked according to the significance of the correlation in CD4^+^ and CD8^+^ can be found as Table S4 in supporting information. Scatter plots depicting the relationship of MZ co-twin differences in DNA methylation and gene expression of TNFSF11 demonstrate the strong correlation in CD4^+^ cells compared to a non-significant correlation in CD8^+^ cells (Figure 3).

**Table 1:** Subset of genes with a correlated difference in DNA methylation and gene expression.

Gene	*ρ*	*P*-value	deltaBeta	log fold change
LDHC	−0.94	0.00000	0.0365	−0.2223
**IL13**	−0.88	0.00000	0.0801	0.5074
**TNFSF11**	0.87	0.00000	−0.0174	−0.5518
MGC3207	−0.86	0.00001	−0.0279	0.1343
CSF2	−0.83	0.00003	0.0455	−1.1684
GIMAP5	−0.82	0.00006	0.0238	−0.5775
GIMAP1	−0.82	0.00007	−0.0092	0.6169
**PTHLH**	0.80	0.00013	−0.0232	−0.0846
DRD1	−0.78	0.00019	−0.0110	−0.1592
NPL	0.78	0.00024	−0.0172	0.2069
EDARADD	−0.77	0.00027	0.0397	0.3906
TCP11L1	0.77	0.00029	−0.0137	0.1352
LIME1	−0.76	0.00037	−0.0182	0.7371
TNFRSF9	−0.76	0.00038	0.0387	0.3343
GSTT1	−0.76	0.00042	−0.0261	0.3210
PHEX	−0.75	0.00047	−0.0626	0.3944
CCL1	−0.75	0.00054	−0.0244	0.5671
FLI1	0.74	0.00060	0.0057	0.1806
SHKBP1	−0.74	0.00067	−0.0133	−0.1847
ARHGAP25	−0.74	0.00068	0.0154	−0.1934
ZNF622	−0.74	0.00073	−0.0079	0.1074
CTSH	−0.74	0.00073	0.0185	0.3537
ANP32E	0.74	0.00076	−0.0058	0.1517
CLSTN3	0.73	0.00078	−0.0071	0.1761
LILRB1	0.73	0.00087	−0.0250	−0.2038
SCN4A	−0.73	0.00088	−0.0174	0.1703
OSBPL7	−0.73	0.00088	−0.0141	0.2682
DDX43	−0.73	0.00091	−0.0571	0.2579
BCL9	−0.72	0.00100	−0.0069	−0.1444
JAK3	0.72	0.00101	0.0384	0.1590
NVL	−0.72	0.00105	0.0119	−0.1287
SFRS5	−0.72	0.00106	0.0064	0.1180
SLAMF7	−0.72	0.00108	−0.0559	0.3104
LOC202459	0.72	0.00114	−0.0143	−0.1368
CYP27B1	0.72	0.00116	−0.0151	−0.2099
KIAA0652	−0.72	0.00120	−0.0292	−0.1276
MRPL49	0.71	0.00127	−0.0107	−0.1856
CTSW	−0.71	0.00135	−0.0354	−0.3207
FLJ36116	−0.71	0.00140	−0.0073	−0.1259
HIST1H1B	−0.71	0.00145	0.0109	0.3023
CD53	−0.71	0.00145	0.0156	0.3137
SAMD10	−0.71	0.00147	−0.0218	0.2655
SPESP1	−0.70	0.00181	−0.0804	0.2557
CTNNA1	0.70	0.00183	−0.0201	−0.1741
HLA-DPA1	−0.70	0.00189	−0.0235	−0.3164
LPIN2	−0.69	0.00201	−0.0174	0.2089
NLN	0.69	0.00202	−0.0388	−0.2258
DPM1	0.69	0.00203	0.0216	−0.1278
MEF2C	0.69	0.00207	−0.0100	0.1793
CCL3L1	0.69	0.00215	−0.0318	−1.0990
HRIHFB2122	−0.69	0.00221	0.0261	0.2449
**ALOX5AP**	−0.69	0.00237	−0.0402	0.6443

This table consists of the top 50 genes ranked according to the significance of the correlation of differences in DNA methylation and gene expression between unaffected and affected MZ co-twins in CD4^+^ cells (genes known to be associated with psoriasis are shown in bold). The magnitude of the mean differences in DNA methylation and gene expression (unaffected versus affected) are presented as deltaBeta and log fold change, respectively.

We used DAVID [31], [32] to explore the potential of shared biological pathways among the genes in the list we generated from the combined analysis of DNA methylation and gene expression in CD4^+^ and CD8^+^ cells (Table S4). GO analyses identified significant enrichment of GO terms in CD4^+^ cells (Table 2), whereas the analysis did not detect any enriched terms in CD8^+^ cells. A significant portion of the top 1% of the genes ranked at the top of the list were found to be involved in the immune response (GO: 0006955, 12.5%, *P* = 0.042), positive regulation of response to stimulus (GO: 0048584, 7.5%, *P* = 0.037), immune system process (GO: 0002376, 15%, *P* = 0.043) and regulation of response to stimulus (GO: 0048583, 10.8%, *P* = 0.043). All of these categories contain genes involved in the immune response, which are potentially important in autoimmune diseases. Interestingly, a subset of the genes identified in the GO analysis (IL13, IL23R, CCL1, CCL5, CSF2, TNFSF11, LTB and SF9) comprises part of the cytokine-cytokine receptor interaction pathway, which is essential in communication between cells in the immune system. Skewed cytokine levels of pro-inflammatory and anti-inflammatory cytokines characterize the pathogenesis of psoriasis. Cytokines and chemokines are essential in the communication between cells in the immune system. Whereas cytokines generally influence proliferation, differentiation and secretion of pro- or anti-inflammatory factors, chemokines primarily have an effect on the movement of cells [33]. Thus, pathways like the cytokine-cytokine receptor interaction are indeed relevant in the etiology of psoriasis. In this context, our results suggest that DNA methylation is important in regulation of the cytokine cascade and signaling pathways involved in psoriasis.

**Table 2:** Gene ontology results of significantly enriched GO terms identified in a combined analysis of DNA methylation and gene expression in CD4^+^ cells.

Category	Term	Count^a^	Frequency (%)^b^	*P*-value^c^
GO:0048584	Positive regulation of response to stimulus	9	7.5	0.037
GO:0006955	Immune response	15	12.5	0.042
GO:0048583	Regulation of response to stimulus	13	10.8	0.043
GO:0002376	Immune system process	18	15	0.043

a Number of genes in the given input gene list which are involved in a specific GO term.

b Percentage of the genes in the given input gene list which are involved in a specific GO term.

c Adjusted according to Benjamini and Hochberg.

Both CD4^+^ and CD8^+^ cells are known to be present in psoriatic plaques, and current evidence indicates that CD4^+^ cells play a more critical role than CD8^+^ cells [34]. Our data strongly suggest that CD4^+^ cells are important in the pathogenesis of psoriasis. However, in this context it is important to recognize the complexity of CD4^+^ cells, which consists of several subpopulations with specific roles in the immune system (i.e. upon activation, naïve CD4^+^ cells develop into several lineages; T_h_1 cells, T_h_2 cells, T_h_22 and T regulatory cells). Recently, much attention has been drawn towards T_h_17 cells and the role in the pathogenesis of psoriasis [35]. It has also been speculated that CD4^+^ cells at different differentiation states may be present, which complicates the picture even further [36]. Distinctive compositions of these subpopulations can potentially contribute to the observed intra-pair differences. In addition, the complexity of the CD4^+^ and CD8^+^ cells could explain the small intra-pair differences by averaging out the level of DNA methylation and gene expression. Thus, a disease-associated change in DNA methylation and gene expression in a subset of cells can ultimately appear as an overall small difference.

Recently, several genes and pathways associated with psoriasis have been identified in GWAS [7]–[13]. Many of these have an essential role in the immune system and this clearly demonstrates the importance of immune response regulation in the disease. The molecular mechanisms driving the inflammation in skin causing psoriasis are complex. Our findings identify new potential susceptibility genes and point to different plausible biological pathways in psoriasis that are under epigenetic regulation and suggest an epigenetic dysregulation of biological pathways implicated in immune function. It will be important to expand on these findings in larger twin and other non-twin cohorts.

## Materials and Methods

### Twin Collection

The twins were recruited pair-wise from the Norwegian Twin Registry (NTR). Altogether 105 pairs were invited to participate, 60 pairs were invited from the cohorts born 1967–1979 [37], [38] and 45 pairs were invited from the cohorts born 1924–1960 [39]. The selection of discordant MZ pairs was based on a two-step procedure. Initial screening was conducted using self-reported data collected via questionnaires in earlier studies [37]–[39]. Pairs for which both twins consented to participate were called in to a clinical dermatology interview and skin examination at Oslo University Hospital, where additional information was also collected. Among the 105 pairs invited through the initial screening phase, 35 pairs consented and 27 pairs clinically evaluated to be discordant for psoriasis participated. The affected twins generally presented with a mild form of psoriasis, mainly affecting the scalp, knees and elbows. Scores for body surface area affected (BSA) were generally low and less than 10% for all the affected. The study was approved by the regional ethical committee and written informed consents were obtained.

### Cell Separation

Lymphocyte subpopulations were isolated in a semi-automated way. Briefly, 60 ml EDTA-blood was diluted 1∶1 with RPMI, split into three aliquots and layered over Lymphoprep solution (Axis-Shield) in 50 ml centrifugation tubes. After centrifugation, PBMCs formed a distinct band that was harvested and washed twice to remove contaminating platelets. CD4^+^ cells and CD8^+^ cells were then sequentially isolated using positive and negative isolation kits from Miltenyi on an autoMACS Pro separator (Miltenyi). CD8^+^ cells were positively isolated using CD8^+^ MicroBeads and the Possel program. CD4^+^ cells were then separated from the negative fraction by negative isolation (i.e. by labeling all other cells but the CD4^+^ cells) using CD4^+^ T Cell Isolation kit II and the Deplete program.

### Zygosity Testing

The zygosity for all twin pairs was determined based on 13 microsatellites on chromosomes 13, 18, 21, X and Y.

### Cell Culturing

CD4^+^ cells were cultured for 7 days in X-VIVO (Lonza), exogenous rIL-2 (10 ng/µl) and Dynabeads CD3/CD28 T Cell Expander (Invitrogen) using ½ bead per cell. CD8^+^ cells were cultured for 14 days under the same conditions.

### DNA Isolation and RNA Isolation

DNA was isolated from cultured cells on a Gentra autopure LS (Qiagen) using the 2–5×10^7^ protocol. This resulted in high yields of pure DNA with an A_260_-A_280_ between 1.7 and 1.9. Total RNA was prepared manually from cultured CD4^+^ and CD8^+^ lymphocytes using RNAqueous Small Scale phenol-Free Total RNA Isolation Kit (Ambion) according to manufacturers instructions. RNA quality was checked using an Agilent 2100 Bioanalyser (Agilent Technologies).

### DNA Methylation and Gene Expression Analysis

DNA methylation status was assessed using the Infinium HumanMethylation27 BeadChip, according to manufactures instructions (Illumina). These arrays enabled detection of the methylation status of 27,578 individual CpGs predominantly distributed in the promoters of 14,475 coding genes throughout the genome. The fluorescence data were analyzed in BeadStudio (Illumina) to determine the β-values (quantitative measurement of the methylation) for each CpG and normalized in Bioconductor *lumi* package [40]. We selected 26,690 probes that unambiguously mapped to the genome (hg18) with up to 2 mismatches as was done in Bell et al. [41]. In DNA methylation analysis, we excluded 1 out of 18 pairs in CD4^+^ cells from all analysis due to low bisulfite conversion.

Gene expression profiling was performed using the HumanHT-12 v3 Expression BeadChip, targeting >25,000 annotated genes, according to manufactures instructions (Illumina). The data were quartile normalized in BeadStudio (Illumina).

### Statistical Analysis

All statistical tests were conducted in R (http://www.r-project.org/). The significance of the differences in DNA methylation between CD4^+^ and CD8^+^ cells were calculated based on a paired *t*-test in unaffected twins with overlapping data from both cell-types. Correlation of DNA methylation in discordant MZ co-twins was computed based on a nonparametric Spearman rank correlation. To search for differentially methylated genes between unaffected and affected twins we used a paired *t*-test on the mean β-value on all CpG sites associated with each gene. In addition, we also searched for differentially methylated CpG sites based on the β-value per CpG separately. The FDR significance thresholds were calculated using *stats* (R package), after raw *P*-values of a paired *t*-test had been computed. Nonparametric permutation tests were also performed, where *P*-values were calculated by comparing the *t*-statistic of the true data set with the *t*-statistics resulting from permutations of the affection status of the twins in all possible combinations. The results from these permutation tests produced similar results.

To search for differentially expressed genes between CD4^+^ and CD8^+^ cells and between unaffected and affected twins, the data was first log2 transformed and an empirical Bayes moderated *t*-test was then applied using the Limma package [42]. Correlation of gene expression between MZ co-twins was computed based on the parametric Pearson correlation. All statistical tests were done two-tailed and a false discovery rate (FDR) below 5% was considered significant.

Of the genes represented on the HumanMethylation27 BeadChip, 11,933 were also present on the HumanHT-12 Expression BeadChip, which enabled integrated analysis of the methylation status and gene expression. In the combined analysis of DNA methylation and gene expression, we compared the differences (between co-twins) in mean β-values for the CpGs associated with each gene, with the log fold changes of the gene expression. Using Spearman's rho as a measure for correlation, we then ranked the genes according to the significance of the correlation coefficients.

### GO Analysis

GO analysis was conducted using the DAVID functional annotation tool [31], [32] with the 120 most significantly correlated genes as input (1%) and the 11,933 genes that was used in the combined analysis of DNA methylation and gene expression as background. Analysis was done with default parameters and results corrected for multiple testing by the method of Benjamini and Hochberg [43].

## Supporting Information

Figure S1 Scatter plots of DNA methylation β-values. Upper panel shows scatter plots of DNA methylation for 4 MZ twin pairs. Lower panel shows scatter plots of DNA methylation for 4 randomly selected pairs of unrelated individuals, matched for age and sex.
(TIFF)

Figure S2 Empirical cumulative distribution functions for technical and biological DNA methylation differences per CpG site. The distribution of technical (black) and biological (red) DNA methylation values are significantly different (Kolmogorov-Smirnov, two-sided, *P*-value<2.2×10^−16^).
(TIFF)

Table S1 Genes showing cell-type specific DNA methylation. This table consists of probes on the 27K Infinium BeadChip and corresponding adjusted *P*-values from a paired *t*-test of CD4+ versus CD8+ cells in 12 unaffected individuals.
(XLSX)

Table S2 Genes showing cell-type specific gene expression. This table consists of the genes on the HumanHT-12 v3 Expression BeadChip and corresponding adjusted *P*-value from comparisons of CD4^+^ versus CD8^+^ cells in 13 unaffected individuals.
(XLSX)

Table S3 Genes known to be associated with psoriasis. This table consists of genes identified in GWAS and listed in the OMIM (Online Mendelian Inheritance in Man) database as associated with psoriasis. Only genes which are included on the DNA methylation and gene expression BeadChips are listed. We used this list when performing the Fisher's exact test to search for overrepresentation of genes known to be associated with psoriasis among the top 50 genes ranked according to the significance of the correlated differences in DNA methylation and gene expression between unaffected and affected co-twins.
(XLS)

Table S4 Results from the combined analysis of differences in DNA methylation and gene expression. List of the 11,933 genes and correlation coefficients (*ρ*) from a combined analysis of MZ co-twin differences in DNA methylation and gene expression in CD4^+^ and CD8^+^ cells.
(XLSX)
