# Supplementary figures and images for: DNA Methylation and Gene Expression Changes in Monozygotic Twins Discordant for Psoriasis: Identification of Epigenetically Dysregulated Genes
Source: PLoS Genet. 2012 Jan 19;8(1):e1002454. doi: 10.1371/journal.pgen.1002454 (PMC3262011; doi:10.1371/journal.pgen.1002454)

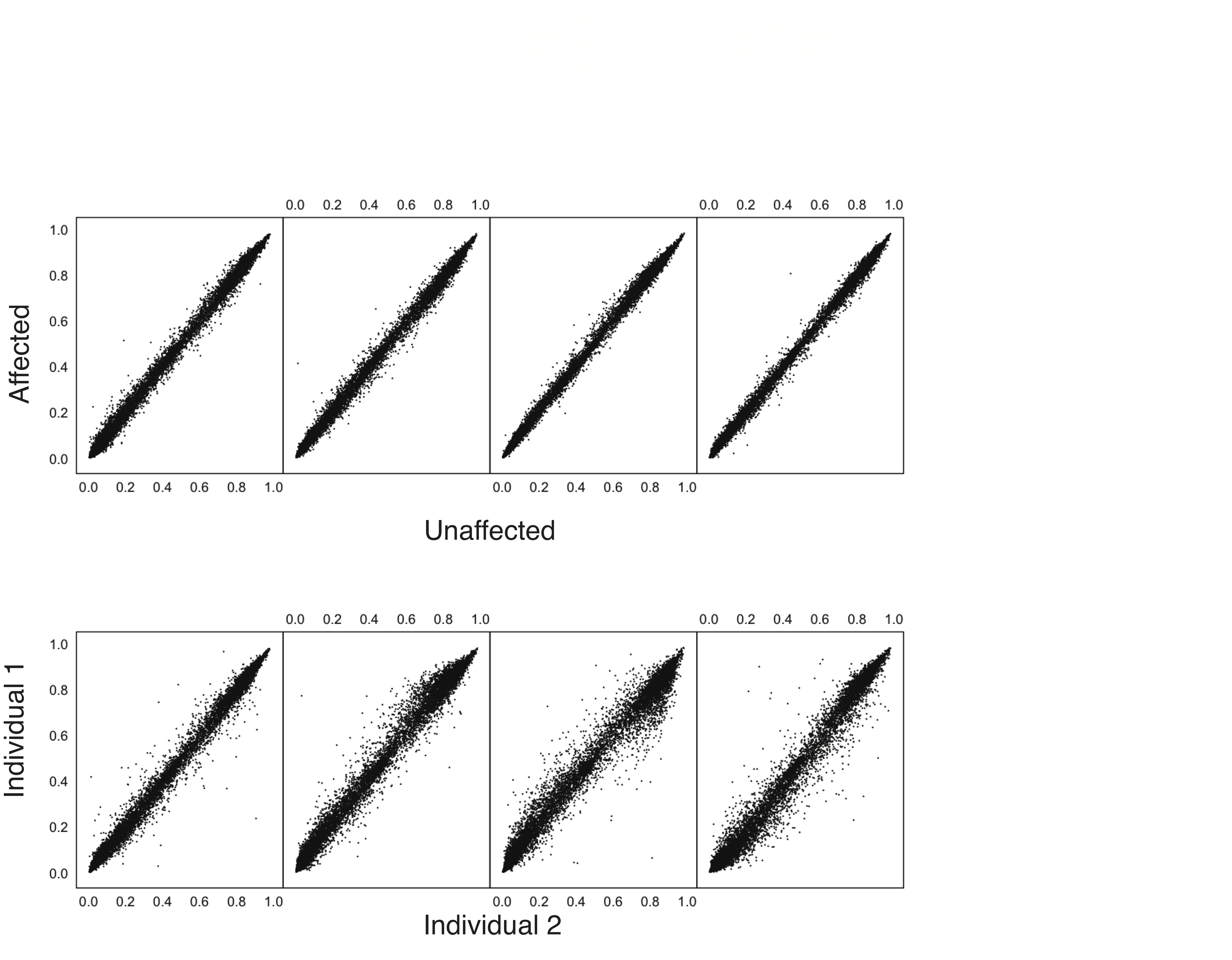

Supplement: Figure S1 — Scatter plots of DNA methylation β-values. Upper panel shows scatter plots of DNA methylation for 4 MZ twin pairs. Lower panel shows scatter plots of DNA methylation for 4 randomly selected pairs of unrelated individuals, matched for age and sex. (TIFF) [file pgen.1002454.s001.tif]
